# Distinct Functions of *Period2* and *Period3* in the Mouse Circadian System Revealed by *In Vitro* Analysis

**DOI:** 10.1371/journal.pone.0008552

**Published:** 2010-01-01

**Authors:** Julie S. Pendergast, Rio C. Friday, Shin Yamazaki

**Affiliations:** Department of Biological Sciences, Vanderbilt University, Nashville, Tennessee, United States of America; Centre National de la Recherche Scientifique, France

## Abstract

The mammalian circadian system, which is composed of a master pacemaker in the suprachiasmatic nuclei (SCN) as well as other oscillators in the brain and peripheral tissues, controls daily rhythms of behavior and physiology. Lesions of the SCN abolish circadian rhythms of locomotor activity and transplants of fetal SCN tissue restore rhythmic behavior with the periodicity of the donor's genotype, suggesting that the SCN determines the period of the circadian behavioral rhythm. According to the model of timekeeping in the SCN, the *Period (Per)* genes are important elements of the transcriptional/translational feedback loops that generate the endogenous circadian rhythm. Previous studies have investigated the functions of the *Per* genes by examining locomotor activity in mice lacking functional PERIOD proteins. Variable behavioral phenotypes were observed depending on the line and genetic background of the mice. In the current study we assessed both wheel-running activity and *Per1*-promoter-driven luciferase expression (*Per1-luc*) in cultured SCN, pituitary, and lung explants from *Per2^−/−^* and *Per3^−/−^* mice congenic with the C57BL/6J strain. We found that the *Per2^−/−^* phenotype is enhanced *in vitro* compared to *in vivo*, such that the period of *Per1-luc* expression in *Per2^−/−^* SCN explants is 1.5 hours shorter than in *Per2^+/+^* SCN, while the free-running period of wheel-running activity is only 11 minutes shorter in *Per2^−/−^* compared to *Per2^+/+^* mice. In contrast, circadian rhythms in SCN explants from *Per3^−/−^* mice do not differ from *Per3^+/+^* mice. Instead, the period and phase of *Per1-luc* expression are significantly altered in *Per3^−/−^* pituitary and lung explants compared to *Per3^+/+^* mice. Taken together these data suggest that the function of each *Per* gene may differ between tissues. *Per2* appears to be important for period determination in the SCN, while *Per3* participates in timekeeping in the pituitary and lung.

## Introduction

Circadian rhythms are self-sustained oscillations in physiology and behavior with endogenous periods of approximately 24 hours that can be entrained to environmental cues such as the light/dark cycle and temperature [1]. In mammals, a light-entrainable circadian pacemaker, located in the suprachiasmatic nuclei (SCN) of the anterior hypothalamus, plays a critical role in the generation of daily rhythms. Surgical destruction of the SCN abolishes most circadian rhythms, including rhythmic locomotor activity [2], [3], [4]. Transplants of fetal SCN tissue to SCN-lesioned animals restore rhythmic behavior that is consistent with the periodicity of the donor's genotype [5], [6], [7], suggesting that the SCN controls the period of the circadian behavioral rhythm.

The SCN is not the only endogenously rhythmic structure. *Ex vivo* adrenals, liver, and retinas exhibit circadian rhythmicity [8], [9], [10], [11], [12]. In addition, genes important for circadian timekeeping are expressed not only in the SCN, but also in many peripheral tissues [13], [14], [15], [16], [17]. Following the discovery that immortalized rat embryonic fibroblasts have circadian rhythms of gene expression [18], real-time monitoring of circadian promoter-driven reporters revealed that peripheral tissues and extra-SCN brain regions exhibit circadian oscillations *in vitro* [19], [20], [21]. Consequently, there is now direct evidence that the mammalian circadian system is composed of multiple circadian oscillators: a light-entrainable central pacemaker in the SCN and many oscillators in other regions of the brain and peripheral tissues.

The molecular mechanism of endogenous rhythm generation in the SCN is modeled as interlocking positive and negative transcriptional and translational feedback loops of circadian gene expression [22], [23], [24]. According to this model, BMAL1/CLOCK or BMAL1/NPAS2 heterodimers activate the transcription of the *Period (Per)* and *Cryptochrome (Cry)* genes. As PER and CRY proteins accumulate, they form complexes and directly bind to BMAL1-CLOCK/NPAS2 heterodimers, thereby inhibiting their own transcription. In addition, post-translational modifications regulate the stability and cellular localization of circadian proteins. The mRNAs of three *Period* homologs (*Per1*, *2*, and *3*) are rhythmically expressed in the SCN, yet only *Per1* and *Per2* have been implicated as important negative elements in the feedback loops that generate the endogenous SCN rhythm [14], [15], [17], [25], [26], [27]. *Per1^−/−^* or *Per2^−/−^* mice, for example, have more severe behavioral phenotypes than *Per3^−/−^* mice. The periods of wheel-running activity of *Per1^−/−^* and *Per2^−/−^* mice can be 1.5 hrs shorter than wildtype mice (depending on the line and genetic background) and they sometimes become arrhythmic in constant darkness (DD), while only a 0.5 h period difference between *Per3^−/−^* and wildtype mice has been observed and *Per3^−/−^* mice never become arrhythmic in DD [28], [29], [30], [31], [32]. In addition, *Per1/Per2* double mutants, but not *Per1/Per3* or *Per2/Per3* double mutants, have arrhythmic locomotor activity in DD [28]. While most of these experiments have been performed in mice with mixed genetic backgrounds or congenic with the 129/sv strain, one study found that *Per2^−/−^* mice congenic with the C57BL/6J strain have periods of wheel-running activity that are indistinguishable from wildtype mice and they do not become arrhythmic in DD [33].

The C57BL/6J strain is ideal for analysis of circadian behavior because the mice have high amplitude (robust), consolidated (not dissociated or fragmented) locomotor activity with a stable free-running period in DD [34], [35]. Since the phenotypes of *Per* mutant mice may vary depending on the genetic background, we sought to investigate the circadian phenotypes of wheel-running behavior and of SCN and peripheral tissue explants in *Per2^−/−^* and *Per3^−/−^* mice congenic with the C57BL/6J strain. In our previous study of C57BL/6J *Per1^−/−^* mice, we found that the real-time circadian gene promoter activity rhythm was weak or absent in SCN slices *in vitro* even though the free-running wheel-running activity rhythm was indistinguishable from wildtype mice [36]. Pituitary and lung explants were also arrhythmic in C57BL/6J *Per1^−/−^* mice. In the current study, we assessed wheel-running activity and rhythmicity in the SCN and peripheral tissues in *Per2^−/−^* and *Per3^−/−^* mice congenic with the C57BL/6J strain. Our approach allowed us to compare the behavioral rhythms to the rhythms of explanted tissues of *Per2^−/−^* and *Per3^−/−^* mice with genetic background held constant.

## Results

### Circadian Wheel-Running Behavior of C57BL/6J *Per2^−/−^* and *Per3^−/−^* Mice

**Figure 1:**
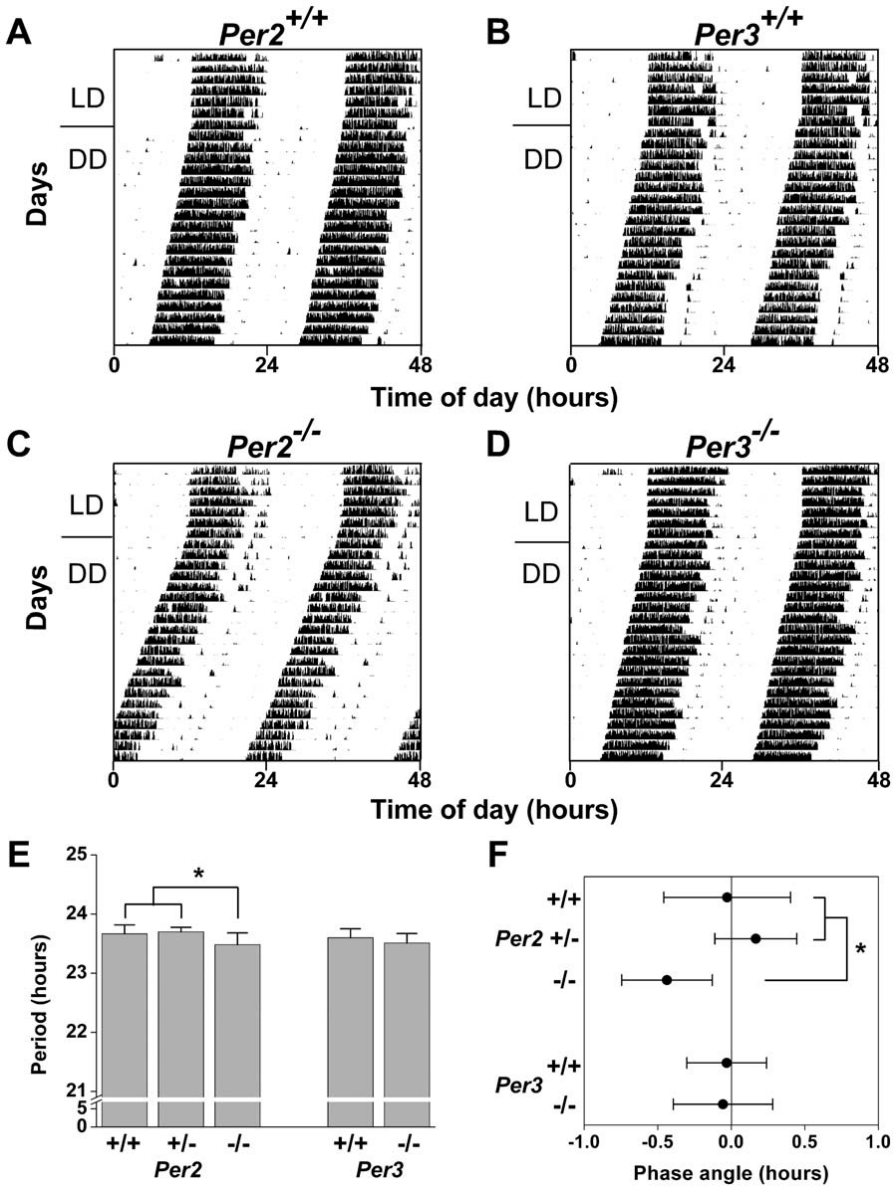
Characterization of Circadian Behavior in C57BL/6J *Per2^−/−^* and *Per3^−/−^* Mice. Representative double-plotted actograms of wheel-running activity of *Per2^+/+^* (A; n = 10), *Per3^+/+^* (B; n = 10), *Per2^−/−^* (C; n = 12), and *Per3^−/−^* (D; n = 10) mice maintained in 12L∶12D LD (lights on at 0 h and lights off at 12 h) for 7 days and then released into constant darkness (DD). The free-running period was determined by using χ^2^ periodogram for days 1-15 in DD (E). The phase angle of entrainment (F) was determined by drawing a regression line to activity onset for days 1-5 in DD and then extending the regression line to the last day in LD. A negative phase angle was obtained when activity started before the time of lights off and a positive phase angle was obtained when activity started after the time of lights off. Data are the mean±SD; *p<0.05.

We generated *Per2^−/−^* and *Per3^−/−^* mice congenic with the C57BL/6J strain by backcrossing *mPer2^−/−^* and *mPer3^−/−^* mutants on an inbred 129/sv genetic background [28], [32] for 10 to 11 generations with C57BL/6J wildtype mice (The Jackson Laboratory, Bar Harbor, ME). We found that *Per2^−/−^* mice had a significantly shorter period of locomotor activity (by ∼11 minutes) compared to *Per2^+/+^* and *Per2^+/−^* mice (*F_2, 28_* = 5.61, *p*<0.01, LSD *p*<0.05; Figure 1A, C, E). In contrast, the period of wheel-running activity in *Per3^−/−^* mice did not differ from *Per3^+/+^* controls (*t_17_* = 1.26, *p* = 0.22; Figure 1B, D, E). The phase angle of entrainment was significantly advanced by 25 minutes in *Per2^−/−^* mice compared to *Per2^+/+^* and *Per2^+/−^* mice (*F_2, 28_* = 8.13, *p*<0.01, LSD *p*<0.05; Figure 1F). The phase angle of entrainment of *Per3^−/−^* mice did not differ from *Per3^+/+^* mice (*t_17_* = 0.18, *p* = 0.86; Figure 1F). Total activity levels and the amplitudes (Q_p_) of wheel-running activity in *Per2^−/−^* and *Per3^−/−^* mice were indistinguishable from wildtype controls (Table 1). The Q_p_ of wheel-running activity in *Per2^+/−^* mice was significantly greater than in *Per2^+/+^* and *Per2^−/−^* mice (*F_2, 28_* = 3.77, *p* = 0.04, LSD p<0.05; Table 1). None of the mice in our study became arrhythmic even after 3 weeks in DD.

**Table 1:** Circadian Behavior of C57BL/6J *Per2^−/−^* and *Per3^−/−^* Mice.

	**Genotype**	**Mean (revs/day)**	**SD**	**n**	***p***
**Activity/day**	*Per2^+/+^*	3726.37	1618.62	9	*F*_2, 28_ = 2.12, *p* = 0.14
	*Per2^+/−^*	4713.71	756.35	8	
	*Per2^−/−^*	3410.36	1558.60	12	
	*Per3^+/+^*	4797.62	931.28	9	*t _17_* = 0.96, *p* = 0.35
	*Per3^−/−^*	4257.75	1428.48	10	
	**Genotype**	**Mean**	**SD**	**n**	***p***
**Q_p_**	*Per2^+/+^*	1793.54	658.27	9	*F*_2, 28_ = 3.77, *p* = 0.04
	*Per2^+/−^*	2466.54	184.55	8	aLSD *p*<0.05
	*Per2^−/−^*	1805.20	685.741	12	
	*Per3^+/+^*	2304.96	423.78	9	*t _17_* = 1.08, *p* = 0.30
	*Per3^−/−^*	2063.37	538.76	10	

a *Per2^+/−^* is significantly greater than *Per2^+/+^* and *Per2^−/−^*.

* The same mice were used to determine period and phase angle of entrainment (Figure 1E, F).

### Lack of Functional PER2 Affects the Circadian Rhythm in C57BL/6J SCN Explants

**Figure 2:**
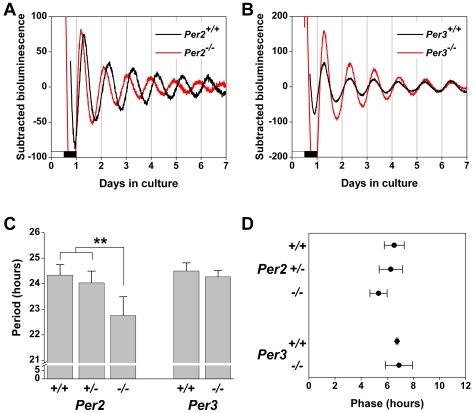
*Per1-luc* Expression in SCN Explants from C57BL/6J *Per2^−/−^* and *Per3^−/−^* Mice. Representative baseline-subtracted bioluminescence rhythms in SCN explants from *Per2^+/+^* (black trace; A; n = 5) and *Per2^−/−^* (red trace; A; n = 6) mice and from *Per3^+/+^* (black trace; B; n = 4) and *Per3^−/−^* (red trace; B; n = 5) mice. The lighting condition of the previous light/dark cycle is indicated for the first day; open bars are light and black bars are dark. (C) The period was determined by fitting a regression line to the acrophase of the *Per1*-*luc* rhythm. (D) The phase was designated as the first peak of *Per1-luc* expression *in vitro* and is plotted relative to the light-dark cycle before culture, where 0 h is the time of lights on and 12 h is the time of lights off (only the subjective day is shown). All data are presented as the mean±SD; **p<0.01.

We next assessed *Per1-luc* expression in cultured SCN explanted from *Period* mutant mice (Figure 2). *Per2^−/−^* SCN explants exhibited robust rhythmicity, but the period of *Per1-luc* expression was significantly shorter than in SCN from *Per2^+/+^* and *Per2^+/−^* mice (*F_2, 14_* = 11.61, *p*<0.01, LSD *p*<0.01; Figure 2A, C). There was also a trend for the phase of the *Per1-luc* expression rhythm in *Per2^−/−^* SCN explants to be advanced compared to *Per2^+/+^* and *Per2^+/−^* SCN (*F_2, 14_* = 3.83, *p* = 0.05; Figure 2D).

*Per1-luc* expression in *Per3^−/−^* SCN explants was indistinguishable from *Per3^+/+^* SCN. The period (*t_6_* = 1.17, *p* = 0.29; Figure 2B, C) and phase (*t_7_* = −0.28, *p* = 0.79; Figure 2D) of *Per1-luc* expression in *Per3^−/−^* SCN did not differ from *Per3^+/+^* SCN.

**Figure 3:**
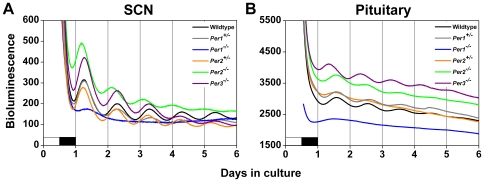
*Per1* Promoter Activity Is Altered in PER-deficient Tissues. Average *Per1-luc* bioluminescence (raw data) from SCN explants (A) and whole pituitary glands (B) from wildtype, heterozygous, or PER-deficient mice. Bioluminescence data from *Per1^−/−^* mice were taken from [36]. The lighting condition of the previous light/dark cycle is indicated for the first day; open bars are light and black bars are dark.

Baseline *Per1* promoter activity in the SCN was also altered by loss of *Per* function. In SCN explants, *Per1* promoter activity was elevated in *Per2^−/−^* and *Per3^−/−^* mice compared to wildtypes and heterozygotes (Figure 3A), suggesting that PER2 and PER3 may inhibit the activation of the *Per1* promoter in wildtype SCN.

### Circadian Rhythms in Pituitary and Lung Explants Are Altered in *Per3^−/−^* Mice

**Figure 4:**
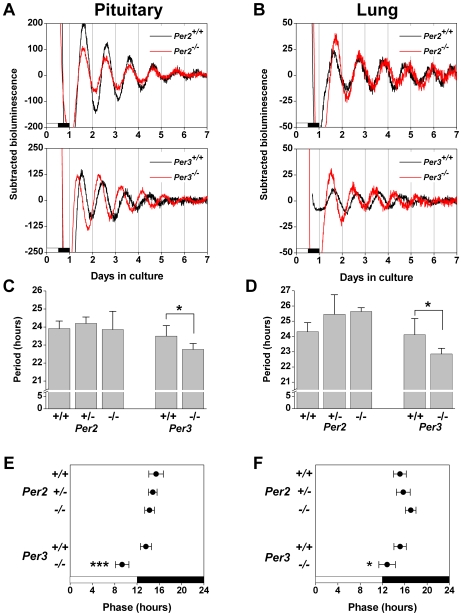
*Per1-luc* Expression in Peripheral Tissues Explanted from C57BL/6J *Per2^−/−^* and *Per3^−/−^* Mice. Representative baseline-subtracted bioluminescence rhythms in pituitary (A) and lung (B) explants from *Per2^+/+^* (black trace; top panel; n = 6) and *Per2^−/−^* (red trace; top panel; n = 4-6) mice and from *Per3^+/+^* (black trace; bottom panel; n = 4) and *Per3^−/−^* (red trace; bottom panel; n = 5) mice. The lighting condition of the previous light/dark cycle is indicated for the first day; open bars are light and black bars are dark. The period of pituitary (C) and lung (D) explants was determined by fitting a regression line to the acrophase of the *Per1*-*luc* rhythm. The phase of pituitary (E) and lung (F) explants was designated as the first peak of *Per1-luc* expression *in vitro* and is plotted relative to the light-dark cycle before culture, where 0 h is the time of lights on and 12 h is the time of lights off (open bars are light and black bars are dark). All data are presented as the mean±SD; *p<0.05, ***p<0.001.

To determine if peripheral tissues have altered circadian rhythms in C57BL/6J *Per*2*^−/−^* and *Per3^−/−^* mice, we assessed *Per1-luc* expression in *ex vivo* pituitary glands and lungs (Figure 4). The period (H = 0.76, d.f. = 2, *p* = 0.68; Figure 4A, C) and phase (*F_2, 16_* = 1.98, *p* = 0.18; Figure 4A, E) of *Per1-luc* expression in *Per2^−/−^* pituitary explants did not differ from *Per2^+/+^* and *Per2^+/−^* mice.

The period of *Per1-luc* expression in *Per3^−/−^* pituitary explants was significantly shorter than in *Per3^+/+^* pituitary (*t_7_* = 2.43, *p*<0.05; Figure 4A, C). In addition, the phase of *Per1-luc* expression was significantly advanced in *Per3^−/−^* pituitary compared to *Per3^+/+^* mice (*t_7_* = 5.69, *p*<0.001; Figure 4A, E).

In pituitary explants, baseline *Per1* promoter activity was elevated in *Per2^−/−^* and *Per3^−/−^* mice compared to wildtypes and heterozygotes (Figure 3B), suggesting that PER2 and PER3 may inhibit the activation of the *Per1* promoter in wildtype pituitary.

There was a trend toward an elongated period of *Per1-luc* expression in lung explants from *Per2^−/−^* and *Per2^+/−^* mice compared to *Per2^+/+^* mice (*F_2, 14_* = 3.84, *p* = 0.05; Figure 4B, D). There was also a trend for *Per2^−/−^* lung explants to have a delayed phase of *Per1-luc* expression compared to *Per2^+/+^* and *Per2^+/−^* lung (*F_2, 14_* = 3.60, *p* = 0.06; Figure 4B, F).

In *Per3^−/−^* lung explants, the period was shorter (*t_7_* = 2.53, *p*<0.05; Figure 4B, D) and the phase of *Per1-luc* expression was advanced (*t_7_* = 2.61, *p*<0.05; Figure 4B, F) compared to *Per3^+/+^* lung.

## Discussion

Previous studies have demonstrated that the SCN controls the period of the circadian locomotor activity rhythm in mammals [7], [37], [38]. Therefore, it is reasonable to predict that if a molecular alteration of the timekeeping mechanism changes the period of the circadian rhythm in the SCN, then the period of locomotor activity should also change (in a similar direction and magnitude). This hypothesis is supported by previous studies in *tau* mutant hamsters and *Clock* (Δ19) mutant mice [39], [40], [41]. In contrast, we found that clock gene promoter-driven luciferase activity was arrhythmic or had low amplitude, irregular rhythms in SCN explants from C57BL/6J *Per1^−/−^* mice even though their locomotor behavior was indistinguishable from wildtype mice [36]. In the current study we assessed locomotor activity and rhythmicity in the SCN of *Per2^−/−^* and *Per3^−/−^* mice to determine if the *in vitro* phenotypes of the mutant SCN were congruent with behavior.

We found that the period of wheel-running activity was 11 minutes shorter and the phase angle of entrainment was advanced by 25 minutes in C57BL/6J *Per2^−/−^* mice compared to *Per2^+/+^* mice. Since we observed only small differences in behavior between *Per2^−/−^* and *Per2^+/+^* mice, we expected to observe a mild phenotype of *Per1-luc* expression in the *Per2^−/−^* SCN. Surprisingly, though, we found that the period of *Per1-luc* expression in the *Per2^−/−^* SCN was 1.5 hours shorter than in the *Per2^+/+^* SCN. The period was shorter in both the SCN and behavior, but the *Per2* mutant phenotype was enhanced in SCN explants. Since the phenotype was more pronounced *in vitro* compared to *in vivo*, we hypothesize that *in vivo* factors such as temperature fluctuations, activity feedback, or coupling to extra-SCN oscillators may compensate for the mutant phenotype.

To our knowledge, our study is the first to analyze real-time circadian rhythms of gene expression in SCN explants from *Per2^−/−^* mice. A previous study found that bioluminescence was arrhythmic or had low amplitude, irregular rhythms in immortalized fibroblasts derived from *Per2^−/−^* mice and infected with a lentiviral construct in which the *Per2* promoter drives the expression of luciferase (*mPer2-dluc*) [42]. SCN explants (or dissociated SCN neurons) were not investigated using this lentivirally-mediated method [42]. In contrast to the dissociated *Per2^−/−^* fibroblasts analyzed by Liu et al. [42], we found that SCN, pituitary, and lung explants from C57BL/6J *Per2^−/−^* mice had robust, rhythmic expression of *Per1-luc*. While the period of *Per1-luc* expression in *Per2^−/−^* SCN explants was significantly shorter than in *Per2^+/+^* SCN, circadian rhythms in *ex vivo* pituitary and lung from *Per2^−/−^* mice were largely unaffected compared to *Per2^+/+^* mice.

In contrast to *Per2^−/−^* mice, wheel-running behavior and rhythmicity in the SCN of C57BL/6J *Per3^−/−^* mice were indistinguishable from *Per3^+/+^* mice. Interestingly, though, we found that the period and phase of *Per1-luc* expression in the pituitary and lung were altered when PER3 was not functional. Our findings in *Per3^−/−^* SCN and lung explants are in agreement with a previous study that assessed the expression of the PERIOD2::LUCIFERASE fusion protein in *ex vivo Per3^−/−^* tissues [42]. Since we also found that circadian rhythms were altered in pituitary explants from *Per3^−/−^* mice compared to *Per3^+/+^* mice, it is possible that *Per3* is important for timekeeping in peripheral tissues. Future studies could assess the physiological outputs of peripheral tissues to determine if the *in vitro* phenotypes of *Per3^−/−^* tissues are present *in vivo*.

Taken together, our data suggest that the function of each *Per* gene may differ between tissues. *Per2* appears to be important for period determination in the SCN, while *Per3* participates in timekeeping in the pituitary and lung.

## Materials and Methods

### Animals

We obtained *mPer2^ldc−/−^* and *mPer3^−/−^* mice [28], [32] (provided by Dr. David Weaver, University of MA, congenic with the 129/sv genetic background) and backcrossed the mice with wildtype C57BL/6J mice (Jackson Laboratory, Bar Harbor, ME) for 10 to 11 generations (C57BL/6J *Per2^−/−^* and *Per3^−/−^* mice are available from The Jackson Laboratory, stock #10492 and #10493, respectively). Heterozygous mice were then crossed with C57BL/6J *Per1-luc* transgenic mice (1-8L) [43] to generate mice that were heterozygous for the *Period* gene and for the *Per1-luc* transgene. *Period* heterozygous (without *Per1-luc* transgene) mice were then crossed with *Period* heterozygous mice with the *Per1-luc* transgene to generate wildtype, heterozygous, and homozygous mutant mice that expressed *Per1-luc* (N10 to N11) that were used for experiments. Genotype was determined by PCR amplification of tail DNA as previously described [28], [32]. The mice were bred and group-housed in the Vanderbilt University animal facility in a 12 h-light/12 h-dark cycle (12L∶12D) and provided food and water *ad libitum*. Male and female mice were used for assessing behavior and for preparing tissue explants. The ages at the beginning of the experiment (mean±SD days) and sexes for each experimental condition are: *Per2^+/+^* behavior: 37.8±10.0, 5 males/5 females; *Per2^+/−^* behavior: 89.3±60.1, 6 males/3 females; *Per2^−/−^* behavior: 49.3±17.2, 8 males/4 females; *Per3^+/+^* behavior: 43.6±9.7, 4 males/6 females; *Per3^−/−^* behavior: 43.1±10.2, 5 males/5 females; *Per2^+/+^* tissue: 66.3±7.7, 3 males/3 females; *Per2^+/−^* tissue: 77.0±9.3, 3 males/2 females; *Per2^−/−^* tissue: 64.3±21.1, 3 males/3 females; *Per3^+/+^* tissue: 113.8±39.9, 3 males/1 female; *Per3^−/−^* tissue: 81.8±22.3, 4 males/1 female. All experiments were conducted in accordance with the guidelines of the Institutional Animal Care and Use Committee at Vanderbilt University.

### Analysis of Wheel-Running Activity

For experiments assessing wheel-running activity, mice were singly housed in cages (33×17×14 cm) with unlimited access to a running wheel (diameter: 11 cm), food, and water. The cages were placed in light-tight, ventilated boxes where the light intensity was 350 lux. Wheel running activity was monitored by a micro-switch-activated signal using the ClockLab system (Actimetrics, Wilmette, IL) and was collected by computer every minute. Analysis was performed using ClockLab software. Free-running period was determined by using a χ^2^ periodogram for 15 days (days 1–15 in DD). The amplitude (Q_p_) of the wheel-running rhythm was the peak value of the χ^2^ periodogram. Total activity level was determined by counting the total number of wheel revolutions from days 1–15 in DD and then averaging them to determine daily activity level. The phase angle of entrainment was defined as the time difference between activity onset and the predicted time of dark onset on the first day in DD. This was calculated by drawing a regression line to activity onset for days 1–5 in DD and then extending the regression line to the last day in LD. A negative phase angle was obtained when activity started before lights off and a positive phase angle was obtained when activity started after lights off.

### Luminescence Recording

The detailed methods for real-time measurement of luminescence from *ex vivo* tissues have been described [44]. Coronal slices of the SCN (300 µm) were prepared by trimming away most extra-SCN tissue and bioluminescence was measured using the LumiCycle apparatus. LumiCycle software (Actimetrics Inc., Wilmette, IL) was used to subtract the 24-hour moving average from the raw luminescence data and to smooth the data by 0.5-hour adjacent averaging. To determine period and phase, the baseline-subtracted and smoothed data was exported to ClockLab (Actimetrics Inc., Wilmette, IL). The period was determined by fitting a regression line to the acrophase of at least 3 days of the *Per1-luc* rhythm and the phase was determined from the first peak of *Per1-luc* expression *in vitro*.

### Statistical Analysis

Statistical analysis was performed using SigmaStat (Systat Software, Inc., San Jose, CA). One-way ANOVA followed by *post-hoc* Fisher's least significant difference (LSD) tests were used for comparison of more than two groups and independent *t* tests (two-tailed) were used to compare two groups except when data was not normally distributed or variances were not homogeneous. The Kolmogorov-Smirnov test (with Lilliefors' correction) was used to test data for normality. For nonparametric analyses, the Kruskal-Wallis One-way ANOVA on Ranks followed by *post-hoc* Dunn's Method were used. Significance was ascribed at *p*<0.05.
